# Factors associated with the severity of hypertension among Malaysian adults

**DOI:** 10.1371/journal.pone.0207472

**Published:** 2019-01-03

**Authors:** Balkish Mahadir Naidu, Muhammad Fadhli Mohd Yusoff, Sarimah Abdullah, Kamarul Imran Musa, Najib Majdi Yaacob, Maria Safura Mohamad, Norhafizah Sahril, Tahir Aris

**Affiliations:** 1 School of Medical Sciences, Health Campus, Universiti Sains Malaysia, Kelantan, Malaysia; 2 Department of Statistics, Federal Government Administrative Centre, Putrajaya, Malaysia; 3 Institute for Public Health, Ministry of Health Malaysia, Kuala Lumpur, Malaysia; Purdue University, UNITED STATES

## Abstract

High blood pressure is a worldwide problem and major global health burden. Whether alone or combined with other metabolic diseases, high blood pressure increases the risk of cardiovascular disease. This study is a secondary data analysis from the National Health and Morbidity Survey 2015, a population-based study that was conducted nationwide in Malaysia using a multi-stage stratified cluster sampling design. A total of 15,738 adults ≥18-years-old were recruited into the study, which reports the prevalence of hypertension stages among adults in Malaysia using the JNC7 criteria and determinants of its severity. The overall prevalence of raised blood pressure was 66.8%, with 45.8% having prehypertension, 15.1% having Stage 1 hypertension, and 5.9% having Stage 2 hypertension. In the multivariate analysis, a higher likelihood of having prehypertension was observed among respondents with advancing age, males (OR = 2.74, 95% CI: 2.41–3.12), Malay ethnicity (OR = 1.21, 95% CI: 1.02–1.44), lower socioeconomic status, and excessive weight. The factors associated with clinical hypertension (Stages 1 and 2) were older age, rural residency (Stage 1 OR = 1.22, Stage 2 OR = 1.28), Malay ethnicity (Stage 2 OR = 1.64), diabetes (Stage 2 OR = 1.47), hypercholesterolemia (Stage 1 OR = 1.34, Stage 2 OR = 1.82), being overweight (Stage 1 OR = 2.86, Stage 2 OR = 3.44), obesity (Stage 1 OR = 9.01, Stage 2 OR = 13.72), and lower socioeconomic status. Almost 70% of Malaysian adults are at a risk of elevated blood pressure. The highest prevalence was in the prehypertension group, which clearly predicts a future incurable burden of the disease. Public health awareness, campaigns through mass and social media, and intervention in the work place should be a priority to control this epidemic.

## Introduction

High blood pressure is a classic worldwide problem and remains a major global health burden. Either alone or combined with other metabolic diseases such as diabetes and obesity, high blood pressure increases the risk of cardiovascular diseases, e.g., stroke and ischemic heart diseases [1,2]. Approximately 40% of adults in the world over the age of 25 have been clinically diagnosed with hypertension [3]. Studies on the clustering of cardiovascular disease burden in 21 regions have shown that more than 9 million deaths were due to the complications of hypertension [4]. This epidemic was also responsible for 7.4 million deaths from coronary heart disease and 6.7 million deaths from stroke [4,5]. Excellent work from the NCD Risk Factor Collaboration on worldwide trends in blood pressure from 1975 to 2015 using 1,479 population-based studies found that the number of adults with hypertension had risen from 594 million in 1975 to 1.13 billion people in 2015 [6]. Furthermore, according to WHO, almost 1.56 billion people (29.2% of the world’s population) are projected to have hypertension by the year 2025 [7].

Hypertension, especially uncontrolled and untreated hypertension, is associated with an increased risk of total and cardiovascular mortality among the general hypertensive population [8]. Extensive research on the effect of potentially modifiable risk factors for myocardial infarction with large-scale data (52 countries) showed that those with severely raised blood pressure have an increased risk of having a myocardial infarction, 2.5 times greater than those with normal blood pressure, regardless of ethnicity, sex, and smoking status [9]. Not only were those with clinical hypertension at risk, those in the prehypertension group were also at least 1.5 times more likely to develop cardiovascular disease compared to those with normal blood pressure [10,11]. Supporting this, a 34-year follow-up of the Framingham Heart Study cohort indicated that the risk of congestive heart failure was more than 2 times higher for those in the higher blood pressure quintile compared to those in the lower quintile at the beginning of the study [12].

Asian countries face the threat of a hypertension epidemic, especially the highly industrialized countries. In the year 2000, over 180 million people in China were estimated to have hypertension, and it was estimated that by the year 2025, this number would increase by about 100 million people. However, a recent study from the NCD Risk Collaboration (2017) highlighted that the current burden was already near that projection. More than the ~40% of the 1.13 billion adults with hypertension lived in Asia in 2015, with 226 million in China alone [6].

Over recent decades, the prevalence of hypertension in Malaysia has remained high, and there has been no significant improvement in the community despite the policies and hypertension task forces implemented by the government [13–15]. Current population-based research in Malaysia shows that hypertension is relatively higher in men, older groups, and those with low household incomes [16]. However, previous studies have only focused on clinical hypertension among adults, with the documentation of factors associated with the severity of hypertension, and most importantly among the vulnerable prehypertensive group, in the local setting being limited. Although prehypertension is not yet considered a disease category, its early detection could prevent future risk for developing hypertension and other cardiovascular diseases [2]. Thus, this study was intended to estimate the prevalence of hypertension by severity and to quantify the associated factors with prehypertension, Stage 1 hypertension, and Stage 2 hypertension. Quantifying the potential risk factors associated with different hypertension stages will help in designing community-based educational and intervention programs for vulnerable populations.

## Material and methods

### Subjects

Adults aged 18 years and above with complete main socio-demographic variables and complete systolic and diastolic blood pressure measurements were included in the study.

### Source of data

The National Health and Morbidity Survey 2015 (NHMS 2015) was a cross-sectional population-based study of the Malaysian population who were non-institutionalized and residing in Malaysia for at least 2 weeks prior to the data collection. The study included a nationally representative sample of urban and rural populations. The sampling design involved a 2-stage probability sampling that accounted for national and subnational estimates. A multistage stratified cluster sampling strategy was utilized in the survey. The country was divided into contiguous geographical areas (clusters) with identified boundaries called enumeration blocks (EBs), which were used as the primary sampling units in this study. These EBs constituted the sampling frame of the NHMS 2015, which was provided by the Department of Statistics (DOS) Malaysia. There were ~75,000 EBs in Malaysia, with 49,000 in urban areas and 26,000 in rural areas. The EBs in the sampling frame were identified as either urban or rural using the classifications provided by DOS Malaysia and based on the population density of the areas. An urban area was defined as a gazetted built-up area with a population of 10,000 or more at the time of the current census (2010) and with 60% of those over age 15 involved in non-agricultural activities, while a rural area was defined as a gazetted built-up area with a population of less than 10,000 [17–18].

The first stage of sampling involved the probabilistic random selection of EBs within the strata. The second stage of the sampling design was the selection of the living quarters from the selected EBs that formed the secondary sampling units. All households and all eligible respondents within a selected living quarter were included in the survey. The study included 30 strata that were formed from the state-urban-rural stratification, and 869 clusters selected (536 urban, 333 rural) from across the country (both Peninsular and East Malaysia).

A bilingual (Malay and English) pre-coded questionnaire was designed and pre-tested with a pilot test prior to the commencement of the survey. Face-to-face interviews were conducted and self-administered questionnaires were handed out in this survey. The clinical data were collected by trained research nurses, and the field data collection was conducted for 3 months from March to June 2015 [17].

### Use of variables

#### Dependent variables

Blood pressure was taken using a calibrated digital automatic blood pressure monitor (OMRON) by trained nurses after 15 minutes of rest with the participants seated. Three readings of the systolic blood pressure (SBP) and diastolic blood pressure (DBP) were taken at 5 minutes intervals with appropriately sized cuffs based on the standard protocol [17].

Following the guidelines from the Seventh Joint National Committee report (JNC7), a subject who has normal blood pressure is defined as having an average SBP of less than 120 mmHg and average DBP of less than 80 mmHg. Individuals with a SBP within the range of 120–139 mmHg and/or DBP within the range of 80–89 mmHg are defined as having prehypertension, the pre-disease category. Clinical hypertension is further categorized as Stage 1 hypertension, defined as SBPs ranging from 140–159 mmHg or DBPs from 90–99 mmHg, and Stage 2 hypertension, defined as a SBP ≥160 mmHg or a DBP ≥100 mmHg [2,15,19]. Participants that refused the blood pressure measurement procedure or who had incomplete/out of range blood pressure readings were excluded from the analysis. Those who had incomplete main sociodemographic profiles, namely age, gender, and residential area, were also excluded from the dataset. Pregnant women were also excluded due to the variability in blood pressure that is common among them [20]. According to the main NHMS 2015 report, 16,017 of 16,681 respondents who claimed they were not previously diagnosed as hypertensive consented to the blood pressure measurement. For the purposes of the current study, 279 (1.7%) of the 16,017 subjects were excluded due to pregnancy or incomplete data [17]. In addition, individuals who were on antihypertensive medication within 2 weeks prior to the visit were also excluded from the analysis. This question was only asked of those who claimed to have had hypertension previously.

#### Independent variables

Information on the demographics, including age, residential location, sex, ethnicity, educational attainments, marital status, and household income status, was collected using a standard questionnaire involving the household and individual levels as published elsewhere and operationalized into suitable groups [17]. Four behavior-related variables were included: (1) drinking status (ex-drinker, has not consumed an alcoholic drink for the past 12 months; non-drinker, never consumed any alcoholic drinks; current drinker, consumed at least 1 standard drink in the past 12 months); (2) current smoker, smokes tobacco product daily or occasionally; (3) fruit and vegetable consumption using the STEPWHO questionnaire (inadequate fruit intake, less than 2 servings per day; inadequate vegetable intake, less than 3 servings per day); (4) physical activity status using the short IPAQ questionnaire: physically active and physically not active according to their Metabolic Equivalent Scores [17,21,22]. Three comorbidity variables were also included: diabetes, hypercholesterolemia, and excessive weight. The subjects who were previously diagnosed by medical professionals to have hypercholesterolemia or diabetes or were diagnosed during the survey were all categorized as having hypercholesterolemia (cholesterol ≥5.1 mmol) or diabetes (fasting blood glucose ≥6.1 mmol or random blood glucose ≥11.1 mmol using a calibrated PA Cardiocheck) [17,23]. Body weight and height were measured to the nearest 0.1 kg and cm, respectively, twice and the calculated body mass indexes (BMI) were categorized according to the WHO 1998 BMI guidelines (underweight, BMI <18.5; normal, BMI 18.5–24.9; overweight, BMI 25.0–29.9; and obese, BMI ≥30) [17,24].

### Statistical analysis

The survey data were analyzed using Stata version 14.0. Complex sample descriptive analyses were utilized to estimate the prevalence. Multivariate multinomial logistic regression modelling was used to examine the effects of the sociodemographic determinants, behavioral factors, and comorbidity factors to determine the potential independent risk factors for hypertension severity. A Wald test was conducted to assess each variable’s contribution to the model. Multicollinearity and interaction were checked accordingly. A final model was created that included all the predictors and interactions that were significantly associated at the level of p <0.05. The overall fitness was checked using a weighted classification table and weighted ROC (receiver operating characteristic) curve for each binary logit model. A regression diagnostic was performed to identify any influential and extreme outliers. The findings are presented as crude and adjusted odds ratios with their 95% confidence intervals. All analyses utilized the complex sampling design to account for the sample weightage and study design properties.

### Ethical approval

Ethical approval was obtained from the Human Research Ethics Committee of the School of Medical Sciences, Universiti Sains Malaysia (JEPeM Code USM/JEPeM/170904097): 9th January 2018 and the Medical Research and Ethics Committee of the Ministry of Health Malaysia (NMMR-17-1989-37492). All accessed data were fully anonymized after permission was obtained to use the NHMS 2015 dataset from the Director General of Health Malaysia on October 2, 2017. The NHMS 2015 main survey and their procedures were reviewed and approved by the NHMS 2015 Steering Community in Kuala Lumpur, Malaysia and the Medical Research and Ethics Committee of the Ministry of Health Malaysia in 2014. Individual written informed consent was obtained from all respondents during the NHMS 2015 data collection [17].

## Results

A total of 15,738 adults were included in the study; the characteristics of the participants are shown in Table 1. The sample was estimated to represent 16.5 million of the Malaysian adult population aged 18 years and older. Of those surveyed, 57.8% resided in urban areas, and the distribution of males and females was almost equal. By ethnicity, 62% were Malay, 22% each were Chinese and Indian, and 15.8% were of other ethnicities. The mean ages of the overall sample, non-hypertension group, prehypertension group, and Stages 1 and 2 hypertension groups were 41.10 (SD ± 15.60), 33.97 (SD ± 13.10), 40.68 (SD ± 14.82), 48.64 (SD ± 15.28), and 53.48 (SD ± 14.40) years, respectively.

**Table 1:** Sociodemographic characteristics of the study sample and the prevalence of prehypertension and hypertension stages 1 and 2 among Malaysian adults.

Variables		Prevalence (95% CI)		
	n (%)	Pre-HPT	HPT Stage 1	HPT Stage 2
**Locality**				
Urban	9094 (57.8)	45.5 (44.14–46.94)	14.5 (13.58–15.46)	5.4 (4.79–5.97)
Rural	6644 (42.2)	46.7 (44.78–48.58)	17.0 (15.62–18.54)	7.6 (6.84–8.48)
**Gender**				
Male	7747 (49.2)	52.6 (51.07–54.15)	16.6 (15.39–17.79)	5.7 (5.05–6.42)
Female	7991 (50.8)	38.1 (36.70–39.61)	13.5 (12.59–14.43)	6.1 (5.55–6.79)
**Ethnicity**				
Malay	9779 (62.1)	45.4 (43.99–46.83)	15.7 (14.78–16.68)	6.9 (6.23–7.57)
Chinese	2383 (15.1)	43.3 (40.62–46.08)	15.2 (13.39–17.31)	5.1 (4.02–6.37)
Indian	1082 (6.9)	47.4 (43.03–51.81)	14.7 (12.01–17.86)	4.6 (3.33–6.36)
Other Bumis^a^	1389 (8.8)	44.5 (41.29–47.82)	14.7 (12.6–17.13)	6.7 (5.34–8.37)
Others^b^	1105 (7.0)	52.9 (48.77–56.93)	12.9 (10.39–15.81)	3.3 (2.36–4.62)
**Educational Attainment**				
Non-formal	918 (5.8)	50.7 (45.98–55.43)	18.6 (15.45–22.18)	15.3 (12.27–19.01)
Primary	3393 (21.6)	47.4 (44.85–49.93)	21.7 (19.83–23.6)	9.9 (8.63–11.39)
Secondary	7524 (47.8)	45.1 (43.52–46.61)	14.6 (13.52–15.72)	4.6 (4.05–5.27)
Tertiary	3710 (23.6)	45.0 (42.93–47.16)	10.9 (9.65–12.36)	3.8 (3.03–4.65)
Others	187 (1.2)	47.3 (38.63–56.04)	14.5 (8.60–23.51)	3.9 (1.30–10.89)
**Marital Status**				
Never Married	3828 (24.3)	44.3 (42.25–46.39)	9.6 (8.38–11.02)	2.8 (2.26–3.57)
Married	10648 (67.7)	46.9 (45.57–48.25)	17.0 (16.06–17.94)	6.7 (6.11–7.40)
Widowed/Divorced	1262 (8.0)	42.0 (38.17–45.96)	24.9 (21.82–28.29)	13.9 (11.6–16.57)
**Income**^c^				
Low	9755 (62.0)	45.5 (44.12–46.97)	16.9 (15.88–17.9)	7.0 (6.33–7.70)
Middle	4410 (28.0)	45.8 (43.84–47.86)	12.9 (11.52–14.34)	4.9 (4.15–5.7)
High	1573 (10.0)	47.0 (43.44–50.63)	12.4 (10.54–14.6)	3.4 (2.45–4.67)
**Current Smoker**				
Yes	3781 (24.0)	52.5 (50.4–54.54)	14.5 (12.94–16.19)	4.6 (3.84–5.55)
No	11955 (76.0)	43.5 (42.19–44.78)	15.3 (14.47–16.23)	6.4 (5.84–6.92)
**Current Drinker**				
Non-Drinker	14110 (93.0)	46.0 (44.75–47.18)	14.9 (14.13–15.8)	6.0 (5.47–6.49)
Ex Drinker	173 (1.1)	41.0 (31.46–51.23)	13.4 (8.59–20.38)	7.4 (2.95–17.28)
Current Drinker	895 (5.9)	45.5 (41.56–49.57)	14.4 (11.95–17.27)	4.5 (3.18–6.36)
**Vegetable Intake**				
Inadequate	14133 (90.1)	45.7 (44.47–46.93)	15.2 (14.42–16.12)	5.9 (5.36–6.39)
Adequate	1550 (9.9)	46.7 (43.43–49.96)	14.2 (12.23–16.4)	6.4 (5.12–7.93)
**Fruit Intake**				
Inadequate	13981 (89.0)	45.5 (44.27–46.67)	15.2 (14.38–16.09)	6.0 (5.47–6.52)
Adequate	1722 (11.0)	49.4 (45.97–52.74)	14.3 (12.3–16.55)	5.3 (4.15–6.74)
**Physical Activity**				
Active	10886 (69.9)	47.6 (46.22–48.94)	15.2 (14.24–16.13)	5.6 (5.03–6.13)
Inactive	4677 (30.1)	41.6 (39.77–43.52)	15.0 (13.7–16.39)	6.8 (5.94–7.77)
**Diabetes Mellitus**				
Yes	2515 (16.0)	43.4 (40.68–46.18)	24.3 (22.06–26.63)	9.6 (8.33–11.12)
No	13223 (84.0)	46.2 (44.95–47.42)	13.7 (12.93–14.53)	5.3 (4.86–5.85)
**Hypercholesterolemia**				
Yes	8110 (51.5)	44.4 (42.91–45.82)	18.8 (17.62–19.95)	8.6 (7.82–9.43)
No	7628 (48.5)	47.1 (45.48–48.75)	11.9 (10.94–12.88)	3.5 (3.05–4.05)
**BMI Status**				
Normal/Underweight^d^	7929 (51.6)	43.6 (41.94–45.22)	10.3 (9.41–11.19)	3.3 (2.89–3.85)
Overweight	4686 (30.5)	50.6 (48.77–52.46)	18.4 (17–19.87)	7.1 (6.16–8.10)
Obese	2750 (17.9)	46 (43.46–48.5)	26.4 (24.31–28.58)	11.7 (10.21–13.44)
**Abdominal Obesity**				
Yes	7624 (49.6)	46.3 (44.8–47.73)	20.8 (19.64–22.05)	9.1 (8.25–9.94)
No	7728 (50.4)	45.8 (44.18–47.49)	10.6 (9.64–11.55)	3.1 (2.62–3.57)

^a^Other Bumis comprising more than 40 indigenous ethnicities that reside in both Peninsular and Borneo, Malaysia

^b^Other ethnicities comprising other Malaysian minorities such as Sikh, Baba, Chitty, Eurasian, and non-citizens

^c^ Low income (< Ringgit Malaysia (RM) 3860), Middle Income (RM 3861–8320), High Income (≥ RM 8321); USD 1 = RM 3.50 in January 2015

^d^ Only 6.5% were underweight

The overall prevalence of hypertension, including prehypertension, among the study population was 66.8% (95% CI: 65.6–68.0), which represented 11,005,000 people based on weighted records. The prevalence of prehypertension was 45.8% (95% CI: 44.66–46.97) followed by 15.1% (95% CI: 14.34–15.92) with Stage 1 hypertension and 5.9% (95% CI: 5.44–6.41) with Stage 2 hypertension. In total, only 33.2% of the population had an optimal blood pressure (SBP <130 mmHg and DBP <80 mmHg), which broke down to 25.1% of the men and 42.3% of the women. The prevalence of prehypertension was higher among men (52.6%) than women (38.1%). The results also revealed that prehypertension was higher in other ethnicities (including minorities such as Sikh, Baba, Chitty, Eurasian, and non-citizens), those with no formal education, married adults, current smokers, and those who were overweight. The prevalence of Stage 1 hypertension was greater among males, residents of rural areas, those with Malay ethnicity, a primary school education, diabetes mellitus, hypercholesterolemia, those who were widowed or divorced, low-income earners, obese, and had abdominal obesity. In contrast, Stage 2 hypertension was more prevalent in females than males (6.1% versus 5.7%). Higher prevalence was also found among those who lived in rural areas, Malay ethnicity, had an informal education, were divorced or widowed, low-income earners, non-smokers, had diabetes mellitus, had hypercholesterolemia, obesity, and abdominal obesity. The details are described in Table 1.

As shown in Table 2, the prevalence of prehypertension increased in both sexes from the youngest age group (43.3%, 95% CI: 41.31–45.39) until the 40–49-year-old age group (49.4%, 95% CI: 47.20–51.64) and then decreased until the oldest age group (40.8%, 95% CI: 37.94–43.63). In contrast, the prevalence of Stages 1 and 2 hypertension increased in each age subgroup for both sexes: Stage 1 hypertension: youngest (7.8%, 95% CI: 6.79–8.95) to oldest (30.5%, 95% CI: 27.88–33.32) age group; Stage 2 hypertension: youngest (1.4%, 95% CI: 1.04–1.91) to oldest (19.0%, 95% CI: 16.72–21.53) age group.

**Table 2:** Age-specific prevalence of prehypertension and stages 1 and 2 hypertension by sex and by residency.

Variables	Prevalence (95% CI)		
	Pre-HPT	Stage 1 HPT	Stage 2 HPT
**All (n = 15,738)**			
**Overall**	**45.8 (44.66–46.97)**	**15.1 (14.34–15.92)**	**5.9 (5.44–6.41)**
18–29 years	43.3 (41.31–45.39)	7.8 (6.79–8.95)	1.4 (1.04–1.91)
30–39 years	48.5 (46.25–50.66)	12.6 (11.22–14.22)	4.0 (3.22–4.91)
40–49 years	49.4 (47.20–51.64)	19.0 (17.33–20.70)	7.5 (6.40–8.89)
50–59 years	46.2 (43.59–48.80)	25.6 (23.50–27.87)	11.6 (10.06–13.45)
≥60 years	40.8 (37.94–43.63)	30.5 (27.88–33.32)	19.0 (16.72–21.53)
**Male (n = 7747)**			
**Overall**	**52.6 (51.07–54.15)**	**16.6 (15.39–17.79)**	**5.7 (5.05–6.42)**
18–29 years	52.5 (49.76–55.18)	11.1 (9.44–13.00)	1.7 (1.11–2.49)
30–39 years	57.3 (54.23–60.34)	14.8 (12.64–17.29)	3.5 (2.58–4.80)
40–49 years	53.9 (50.65–57.09)	19.1 (16.58–21.69)	7.5 (5.83–9.48)
50–59 years	49.0 (45.3–52.61)	24.6 (21.68–27.68)	11.5 (9.14–14.37)
≥60 years	41.3 (37.53–45.13)	29.3 (25.68–33.15)	18.1 (15.04–21.56)
**Female (n = 7991)**			
**Overall**	**38.1 (36.70–39.61)**	**13.5 (12.59–14.43)**	**6.1 (5.55–6.79)**
18–29 years	33.0 (30.48–35.54)	4.1 (3.19–5.17)	1.1 (0.7–1.77)
30–39 years	37.8 (34.94–40.83)	10.0 (8.41–11.95)	4.5 (3.48–5.86)
40–49 years	44.7 (41.61–47.83)	18.9 (16.71–21.33)	7.6 (6.21–9.39)
50–59 years	43.0 (39.66–46.32)	26.9 (23.92–30.06)	11.8 (9.96–13.98)
≥60 years	40.2 (36.23–44.35)	31.8 (28.22–35.63)	20.0 (16.96–23.33)
**Urban (n = 9094)**			
**Overall**	**45.5 (44.14–46.94)**	**14.5 (13.58–15.46)**	**5.4 (4.79–5.97)**
18–29 years	42.0 (39.64–44.44)	7.6 (6.38–8.92)	1.5 (1.06–2.17)
30–39 years	48.6 (45.94–51.24)	12.0 (10.38–13.78)	3.3 (2.49–4.42)
40–49 years	49.2 (46.48–51.87)	18.0 (16.06–20.1)	7 (5.67–8.66)
50–59 years	45.9 (42.77–49.09)	25.1 (22.52–27.84)	10.7 (8.76–12.99)
≥60 years	42.6 (39.03–46.26)	30.3 (26.79–33.99)	17.5 (14.56–20.84)
**Rural (n = 6644)**			
**Overall**	**46.7 (44.78–48.58)**	**17.0 (15.62–18.54)**	**7.6 (6.84–8.48)**
18–29 years	47.2 (43.48–50.93)	8.5 (6.68–10.83)	1.1 (0.66–1.8)
30–39 years	48.0 (44.62–51.37)	15.1 (12.14–18.53)	6.4 (4.85–8.31)
40–49 years	50.3 (46.96–53.56)	22.2 (19.63–25.09)	9.3 (7.48–11.63)
50–59 years	47.0 (42.65–51.33)	27.2 (23.78–30.92)	14.5 (12.17–17.08)
≥60 years	36.5 (32.37–40.92)	31.1 (27.74–34.75)	22.5 (19.39–25.9)

### Factors associated with hypertension stages

The results of the multinomial logistic regression analysis for Malaysian adults are shown in Table 3 with crude and adjusted odds ratios, their 95% confidence intervals, and p-values <0.05. Of the 14 variables included, only alcohol consumption, vegetable intake, and fruit intake were not statistically significant in affecting the chances of having raised blood pressure in the univariate analysis. In the multivariate analysis, individuals at the prehypertensive level showed a gradual increase in their chances of having prehypertension with age, from 40% to 400% relative to those in the youngest age group (18–29-year-olds). Men were 2.5 times more likely to have prehypertension than women (OR = 2.74, 95% CI: 2.40–3.12), and Malays had a greater chance of having prehypertension than Chinese (OR = 1.21, 95% CI: 1.02–1.44). Those with lower educational levels had a 33% to 95% increased odds of having prehypertension compared to those with a tertiary educational level. Overweight (OR = 1.97, 95% CI: 1.74–2.22) and obese (OR = 3.43, 95% CI: 2.89–4.07) individuals had higher odds of prehypertension relative to those with normal weights.

**Table 3:** Factors associated with blood pressure status from univariate and multivariate multinomial logistic regressions (Clusters = 869, Strata = 30, *df* = 839).

Variables	Complex Sample Univariate Multinomial Logistic Regression (Crude Relative Risk Ratio)			Complex Sample Multivariate Multinomial Logistic Regression (Adjusted Relative Risk Ratio)		
	Pre-HPT RRR (95% CI)	HPT Stage 1 RRR (95% CI)	HPT Stage 2 RRR (95% CI)	Pre-HPT ARRR (95% CI)	HPT Stage 1 ARRR (95% CI)	HPT Stage 2 ARRR (95% CI)
**Age Group**						
18–29 years	1	1	1	1	1	1
30–39 years	1.52 (1.33–1.73)**	2.20 (1.78–2.72)**	3.84 (2.63–5.61)**	1.41 (1.19–1.66)**	2.05 (1.6–2.63)**	4.00 (2.65–6.04)**
40–49 years	2.25 (1.95–2.59)**	4.79 (3.88–5.90)**	10.57 (7.3–15.32)**	2.20 (1.83–2.64)**	4.45 (3.46–5.73)**	11.19 (7.40–16.93)**
50–59 years	3.06 (2.57–3.64)**	9.42 (7.51–11.82)**	23.75 (16.43–34.33)**	2.90 (2.32–3.63)**	8.33 (6.22–11.16)**	22.86 (14.71–35.54)**
≥60 years	4.60 (3.73–5.66)**	19.13 (15.03–24.36)**	66.05 (45.13–96.68)**	4.46 (3.37–5.9)**	18.03 (12.93–25.14)**	62.63 (37.72–103.99)**
**Locality**						
Urban	1	1	1	1	1	1
Rural	1.24 (1.11–1.38)**	1.42 (1.22–1.65)**	1.72 (1.43–2.07)**	1.10 (0.97–1.25)	1.22 (1.01–1.47)*	1.28 (1.01–1.62)*
**Gender**						
Male	2.32 (2.11–2.54)**	2.06 (1.81–2.34)**	1.56 (1.31–1.85)**	2.74 (2.41–3.12)**	3.24 (2.73–3.84)**	3.01 (2.36–3.83)**
Female	1	1	1	1	1	1
**Ethnicity**						
Malay	1.19 (1.03–1.38)*	1.17 (0.96–1.42)	1.54 (1.16–2.04)*	1.21 (1.02–1.44)*	1.21 (0.96–1.52)	1.64 (1.17–2.29)*
Chinese	1	1	1	1	1	1
Indian	1.19 (0.95–1.51)	1.05 (0.78–1.42)	0.99 (0.64–1.55)	1.04 (0.81–1.34)	0.84 (0.6–1.17)	0.79 (0.47–1.32)
Other Bumis	1.10 (0.89–1.36)	1.03 (0.78–1.37)	1.41 (0.97–2.05)*	1.1 (0.85–1.41)	1.1 (0.78–1.55)	1.46 (0.92–2.32)
Others	1.43 (1.15–1.78)*	0.99 (0.72–1.35)	0.77 (0.48–1.21)	1.37 (1.06–1.77)*	1.38 (0.94–2.01)	1.22 (0.71–2.1)
**Education**						
Non-formal	2.95 (2.17–4.01)**	4.46 (3.08–6.46)**	10.7 (7.02–16.31)**	1.95 (1.39–2.72)**	1.79 (1.17–2.73)*	3.03 (1.77–5.19)**
Primary	2.01 (1.72–2.37)**	3.79 (3.07–4.69)**	5.06 (3.74–6.84)**	1.33 (1.1–1.63)*	1.60 (1.24–2.06)**	1.66 (1.13–2.43)*
Secondary	1.13 (1.01–1.27)*	1.50 (1.26–1.8)**	1.39 (1.06–1.81)*	0.94 (0.81–1.08)	1.04 (0.85–1.28)	0.84 (0.62–1.13)
Tertiary	1	1	1	1	1	1
Others	1.23 (0.86–1.76)	1.56 (0.83–2.91)	1.2 (0.38–3.81)	1.02 (0.68–1.52)	1.30 (0.63–2.68)	0.99 (0.21–4.67)
**Marital Status**						
Never Married	1	1	1	1	1	1
Married	1.56 (1.4–1.74)**	2.60 (2.17–3.1)**	3.48 (2.66–4.56)**	0.97 (0.84–1.12)	0.81 (0.65–1.01)	0.61 (0.44–0.85)*
Widowed/Divorced	2.14 (1.66–2.76)**	5.84 (4.29–7.95)**	11.04 (7.74–15.74)**	1.12 (0.83–1.5)	1.13 (0.78–1.64)	0.77 (0.5–1.19)
**Income**						
Low	1.18 (0.99–1.39)	1.65 (1.32–2.06)**	2.50 (1.73–3.62)**	1.09 (0.91–1.31)	1.46 (1.11–1.92)*	2.15 (1.36–3.4)*
Middle	0.99 (0.83–1.19)	1.06 (0.82–1.35)	1.46 (0.99–2.15)	0.98 (0.81–1.17)	1.04 (0.78–1.39)	1.53 (0.96–2.44)
High	1	1	1	1	1	1
**Current Smoker**						
Yes	1.48 (1.33–1.65)**	1.16 (0.98–1.37)	0.89 (0.72–1.11)			
No	1	1	1			
**Drinking Status**^a^						
Non-Drinker	1	1	1			
Ex Drinker	0.77 (0.49–1.23)	0.78 (0.44–1.38)	1.07 (0.39–2.96)			
Current Drinker	0.92 (0.76–1.12)	0.9 (0.69–1.16)	0.71 (0.47–1.06)			
**Vegetable Intake**^a^						
Adequate	1	1	1			
Inadequate	0.97 (0.82–1.14)	1.06 (0.86–1.31)	0.9 (0.69–1.19)			
**Fruit Intake**^a^						
Adequate	1	1	1			
Inadequate	0.86 (0.72–1.02)	0.99 (0.79–1.24)	1.05 (0.78–1.41)			
**Physical Activity**						
Inactive	0.76 (0.68–0.84)**	0.86 (0.74–0.99)*	1.06 (0.89–1.27)			
Active	1	1	1			
**Diabetes Mellitus**						
Yes	1.44 (1.23–1.69)**	2.71 (2.29–3.21)**	2.77 (2.23–3.43)**	1.04 (0.89–1.23)0.607	1.47 (1.22–1.78)**	1.2 (0.94–1.51)
No	1	1	1	1	1	1
**Hypercholesterolemia**						
Yes	1.25 (1.13–1.38)**	2.09 (1.83–2.4)**	3.24 (2.71–3.87)**	1.03 (0.92–1.15)0.65	1.34 (1.14–1.56)**	1.82 (1.48–2.25)**
No	1	1	1	1	1	1
**BMI Status**						
Normal/Underweight	1	1	1	1	1	1
Overweight	2.08 (1.85–2.34)**	3.21 (2.74–3.75)**	3.79 (3.07–4.68)**	1.97 (1.74–2.22)**	2.86 (2.41–3.4)**	3.44 (2.74–4.32)**
Obese	2.84 (2.41–3.35)**	6.92 (5.74–8.35)**	9.46 (7.3–12.26)**	3.43 (2.89–4.07)**	9.01 (7.29–11.15)**	13.72 (10.18–18.49)**

The Complex Sample Enter method was used for variable selection. Multicollinearity and interaction were unlikely. Overall fit of the model for each binary logit was checked accordingly: correctly weighted classified table (first binary models, 68%; second binary model,79%; third binary model, 89%), Weighted Area under ROC curve (first binary models, 0.72; second binary model, 0.84; third binary model, 0.72). Models were considered fit based on the classification table and area under the curve. A regression diagnostic was performed, and no influential outliers affected the overall model. Hence, no observations were removed from the model. Final model was adjusted to behavioral variables.

^a^p >0.25 in the univariable analysis;

*p <0.05;

**p <0.001.

At the clinical hypertension level (Stage 1 and 2), the multivariate results suggested that relative to 18–29-year-olds, the odds of having Stage 1 or 2 hypertension for those of advanced age were up to 18 times and 62 times higher, respectively. Rural residents had a 20% greater chance of having both stages of hypertension (Stage 1 OR = 1.22, 95% CI: 1.01–1.47; Stage 2 OR = 1.28, 95% CI: 1.01–1.62) compared to urban residents. Males were 3 times more likely to be hypertensive compared to females for both stages (Stage 1 OR = 3.24, 95% CI: 2.73–1.41; Stage 2 OR = 3.01, 95% CI: 2.36–3.83). The odds for Malays (OR = 1.64 95% CI: 1.17–2.29) having Stage 2 hypertension were significantly higher compared to the Chinese. In addition, compared to individuals with a tertiary education, those without a formal education had higher odds of having Stage 1 (OR = 1.79, 95% CI: 1.17–2.73) and Stage 2 (OR = 3.03, 95% CI: 1.77–5.19) hypertension, while those with a primary school education had a 60% higher chance of Stage 1 and 2 hypertension compared to those with tertiary education. Married people had a 39% lower risk of having Stage 2 hypertension compared to individuals who had never married. Respondents in the low-income bracket had a higher tendency (Stage 1 OR = 1.46, 95% CI: 1.11–1.92; Stage 2 OR = 2.15, 95% CI: 1.36–3.40) to have clinical hypertension compared to those in the higher income bracket. Individuals with diabetes mellitus had 47% higher odds of having Stage 1 hypertension (OR = 1.47 95% CI: 1.22–1.78) compared to non-diabetics. Compared to those with normal cholesterol levels, the odds for individuals with hypercholesterolemia having Stage 1 and 2 hypertension were 1.34 (95% CI: 1.14–1.56) and 1.82 (95% CI: 1.48–2.25), respectively. Increased weight was significantly associated with both stages of hypertension. Relative to those with normal weight, the likelihood of having Stages 1 and 2 hypertension were 2.8 times and 3.4 times higher, respectively, among those who were overweight. Furthermore, obese adults had a 9 times greater chance of having Stage 1 hypertension and almost 14 times greater chance of having Stage 2 hypertension compared to normal-weighted adults.

## Discussion

This nationwide representative study presents the burden of high blood pressure in Malaysia, and it is in a worrisome state. The data indicate that nearly two-thirds of adults in Malaysia are prehypertensive or hypertensive. Age, gender, residence locality, socio-economic status, ethnicity and other comorbidities were shown to affect the likelihood of having raised blood pressure. More precisely, those who were older, male, never married, lived rurally, had a lower socio-economic status, Malay ethnicity, diabetes, hypercholesterolemia, and excessive bodyweight were more likely to have elevated blood pressure levels.

Previous studies have shown that increasing age is associated with higher odds for all stages of hypertension [16,19,25], which was consistent in this study. Similarly, a study conducted among high-income residents found that those in older age groups had more than a 2-fold chance of developing Stage 1 or 2 hypertension and a 34% greater chance of developing prehypertension regardless of other co-existing cardiovascular risk factors [26]. Possible explanations could be that stiffening arteries are associated with the aging process, the lack of physical activity common among the elderly, and a higher sensitivity to salt resulting in increased blood pressure [27–29].

Our study found that those in rural areas were more likely to have more severe hypertension. Similarly, a study from South Africa showed that those living in rural areas were twice as likely to have Stage 2 hypertension compared to urbanites, and a study in China found that rural dwellers had a 3–9 times higher chance of having clinical hypertension [19,30]. In addition, findings from a study in Mexico concluded that rural residents had 5 times higher odds of having uncontrolled hypertension and a 70% lower likelihood of having been treated for hypertension [31]. One possible explanation that might influence this result is that people living in urban areas may be more likely to be treated and have better access to health care [32]. Moreover, studies performed by Wang et. *al*. [30] and Ho *et*. *al* [33] found that urbanites were up to 20% more aware of their high blood pressure status compared to those living in rural areas.

As in our study, men have been found to be more often affected in comparison to women. Chiu *et al*. [34] reported that males had 70% higher odds of progression from a normal blood pressure to prehypertension and 1.2 times higher odds of progression from prehypertension to Stage 1 hypertension. According to Grotto et *al*. [35], among those with high blood pressure, men were more likely to have metabolic syndrome and a sedentary lifestyle compared to women. However, contradictory findings have been reported elsewhere. A large-scale study in Jordan showed that females had 2.5 times higher odds of developing clinical hypertension, and other results from high-income countries have reported a higher proportion (at least 10%) of women with Stages 1 and 2 hypertension [36,37]. Oher reports involving large-scale data sets and a systematic review of Southeast Asian countries were unable to demonstrate a relationship between gender and the development of prehypertension and hypertension (Stages 1 or 2) [19,30,38]. Despite the lack of a clear gender-blood pressure relationship, there is significant evidence that the androgen hormones in men are responsible for influencing the blood pressure regulation differences observed between the genders [39].

Numerous studies have shown an association of high blood pressure with different ethnicities [20,40]. Our study showed that Malays had 21% higher odds of having prehypertension and 64% higher odds of having Stage 2 hypertension compared to the Chinese. The traditional cuisine of Asian people is well known to contain high amounts of salt. According to Lee and Kim [41], salt has been widely used in fish fermentation, pickling, and the production of local Asian sauces, and previous research has established that excessive sodium intake leads to uncontrolled blood pressure among adults [40,42]. A cross-sectional study performed by Rashidah A e*t al*. [43] involving 471 respondents (>90% of Malay ethnicity) showed that the mean sodium intake of both male and female subjects exceeded the recommended amount by at least 70%. However, an earlier study using nationwide data showed no differences in the prevalence of hypertension among the major ethnicities [16].

Several studies have highlighted the relationship between lower socio-economic status and hypertensive individuals. Our study demonstrated that those with less education and lower incomes tended to have more severe hypertension. Studies undertaken in middle-income countries with subjects ≥40-years old found that compared to those with high incomes, those with lower household income were 60% more prone to having Stages 1 and 2 hypertension [31]. In the same vein, in Thailand, a study by Lwin *et al*. [38] showed that those who only attended primary school or had lower levels of education had ~8-fold higher odds of having hypertension compared to those with a high school or higher education. Likewise, findings from Chiu *et al*. [34] emphasized that those with lower educational levels had a 36% higher chance of progressing from Stage 1 to Stage 2 hypertension. However, contrary to our findings, a population-based cross-sectional study in Ghana found that those in the higher income quintile (richer) had 2 times greater risk of having prehypertension and Stages 1 and 2 hypertension relative to the lowest income quintile. According to WHO, those with lower socio-economic status have a higher risk of developing mental health problems such as stress and depression, which could lead to high blood pressure [44]. In addition, the Malaysian Adults Nutrition Survey in 2014 found that those with lower socio-economic status were more prone to eat at food stalls, which are comparatively cheaper and well known for foods with higher salt content [45].

While other studies have not shown an association between marital status and high blood pressure in multivariable analyses, interestingly, our findings showed that those who were married had 14% lower odds of having Stage 2 hypertension [19,36,46]. According to the NHMS 2015 main report, individuals who had never married had a higher prevalence of mental illness (depression, anxiety, and stress) compared to those who were married, which could increase their blood pressure [27]. Lwinn and colleagues [38] established that people with mild to high stress levels were expected to have a more than 2-fold higher likelihood of developing hypertension compared to those not experiencing stress in their lives.

As expected, a significant relationship was seen between diabetes mellitus and hypertension severity. Our study found that those with diabetes mellitus had almost 50% more risk of having Stage 1 hypertension. In agreement with this, past research has demonstrated a positive association between blood glucose abnormalities and blood pressure [11,25,30,35,47]. Moreover, evidence from the Strong Heart Study database, which involved 4,549 adults, showed that the risk of developing more severe high blood pressure was almost 2-fold higher among those with diabetes mellitus. In addition, the same study also observed that those with high insulin resistance had at least a 40% higher risk of developing more severe hypertension compared to those with lower insulin resistance [11]. The elevated blood pressure may be explained by higher macroalbuminuria and microalbuminuria, which Wang *et*.*al* (2006) concluded occurs mainly in individuals with diabetes mellitus, and/or microvascular damage due to chronic hyperglycemia as Awoke *et al*. [11,46] concluded. Complications among diabetes patient, including neuropathy, resistance to treatment, and a high pulse rate, cause difficulties in lowering their blood pressure compared to non-diabetics [48,49].

In accordance with previous studies, a positive association was seen between lipid abnormalities and blood pressure [30,35,37,40,46]. In our study, high blood cholesterol levels significantly increased the chances of having clinical hypertension (from 30% to 80%) compared to those with normal blood cholesterol levels. Similarly, a large scale cohort study established that more than 20% of adults aged 30 years and older with Stage 1 hypertension progress to Stage 2 [34]. One possible explanation could be that the population surveyed had pre-existing high cholesterol levels. Evidence from a cohort study by Shishani *et al*. [37] concluded that the risk of developing clinical hypertension was more than 2-fold higher in the presence of pre-existing hypercholesterolemia. Furthermore, Pereira et *al*. [50] have shown that the decrease in nitric oxide (NO) in those with high plasma lipid levels was an important factor that contributed to the elevated arterial blood pressure.

In our study, being overweight and obese emerged as having the most impact and correlated to more severe hypertension. The findings highlighted that a person in the prehypertension group who was overweight was at least twice as likely to be affected and the odds increased with severity, with obese individuals nearly 14 times as likely to have Stage 2 hypertension. A parallel pattern has been found in a large number of literature reviews [16,19,25,34,38,49]. Previous studies have noted that obese individuals had 11-fold greater odds of having Stage 1 hypertension than individuals of normal weight. In addition, a study in South Africa reported that those who were overweight had more than twice the likelihood of having Stage 1 hypertension and more than 3 times higher chance of having Stage 2 hypertension [19]. Supporting this finding, a study performed in Copenhagen showed that for each 10% increase in BMI, systolic blood pressure increased by 3.85 mmHg, while diastolic blood pressure increased by 1.79 mmHg [51]. Numerous studies have suggested that the long-term effects of weight reduction could lower the likelihood of having high blood pressure. Thus, front line therapy should consist of a modification in lifestyle by increasing physical activity and following a healthy diet regimen [52,53].

In conclusion, this study highlights an alarming situation regarding the prevalence of high blood pressure in Malaysia. Two out of three adults were suffering from high blood pressure, affecting more than 11 million adults aged 18 years and older. The highest prevalence levels were in the prehypertension group, which clearly suggests a future incurable disease burden. A multivariate multinomial regression analysis revealed that increasing age, rural residency, being male, having Malay ethnicity, lower socio-economic status, never having been married, diabetes mellitus, hypercholesterolemia, and excessive weight were factors that increased the likelihood of having more severe hypertension. These findings provide evidence-based information for relevant stakeholders and policy makers in planning and implementing national strategic intervention programs.

### Limitations

Our findings must be considered within the context of the study limitations. The prevalence of hypertension by stages in this study may have been overestimated, as the BP was only measured during a single visit. According to both JNC7 and Malaysia CPG, a blood pressure classification should be defined based on the average of at least 2 or more BP readings taken at 2 or more visits after an initial screening [2,15]. In addition, since the BP measurement was taken by trained nurses dressed in full uniform, there is the possibility of a “white coat effect” happening during the survey, which could also contribute to overestimating the true prevalence [54]. Furthermore, the design of the cross-sectional study cannot be used to establish conclusive cause and effect between the factors and outcome. The data in the main survey were originally collected for a different objective, thus the secondary data that was extracted from the NHMS 2015 was limited to exploring possible associations with the risk of having hypertension and its severity, i.e., genetic factors, family history, dietary factors (sodium intake), and clinical parameters such as blood and urine samples. Finally, most of the information was based on self-reported data, which is subject to recall bias. Despite these limitations, the same field methodology has been used and accepted in large epidemiological studies across the globe [19, 35, 37, 54].

## Supporting information

S1 File (PDF)

S1 Data (DTA)
