# Supplementary material for: Factors associated with the severity of hypertension among Malaysian adults
Source: PLoS One. 2019 Jan 3;14(1):e0207472. doi: 10.1371/journal.pone.0207472 (PMC6317782; doi:10.1371/journal.pone.0207472)
Supplement: S1 File — (PDF) [file pone.0207472.s001.pdf]

TINJAUAN KEBANGSAAN KESIHATAN DAN MORBIDITI 2015

NATIONAL HEALTH AND MORBIDITY SURVEY 2015

BORANG SOAL SELIDIK INDIVIDU
INDIVIDUAL QUESTIONNAIRE

RESPONDEN 13 TAHUN DAN KE ATAS
RESPONDENT 13 YEARS AND ABOVE

INSTITUTE FOR PUBLIC HEALTH

UNTUK DIISI OLEH PENEMURAMAH

Negeri : ____________________

ID : NEGERI ☐☐ DP ☐☐ DB ☐☐☐ BP ☐☐☐☐ STRATA ☐ TK ☐☐☐ ISI RUMAH ☐☐

ID Penemuramah : ☐☐☐☐ **ID Jururawat :** ☐☐☐☐

Keputusan : 1. Lengkap 2. Tidak Lengkap* 3. Enggan Jawab*
4. lain-lain* * Nyatakan ____________________

ISI KANDUNGAN / *CONTENT*

Bil / *No*	Kandungan / *Contents*	Muka Surat / *Page*
A2	Sosiodemografi<br>*Sociodemography*	2
AC	Persepsi Terhadap Penyampaian Sistem Kesihatan<br>*Perception On Healthcare Delivery Systems*	7
A23	Pembayar Perkhidmatan Kesihatan<br>*Payer For Health Services*	11
B1	Laporan Penyakit<br>*Reported Illness*	12
B2	Penggunaan Kemudahan Servis Kesihatan<br>*Health Service Utilisation*	13
B3	Jagaan Kesihatan Mulut Atau Gigi<br>*Dental Care*	18
B4	Jagaan Kesihatan Pesakit Luar<br>*Out-Patient Care*	22
C	Kencing Manis<br>*Diabetes Mellitus*	26
D	Tekanan Darah Tinggi<br>*Hypertension*	28
E	Paras Kolesterol Tinggi<br>*Hypercholesterolaemia*	30
F	Aktiviti Fizikal<br>*Physical Activity*	32
G	Amalan Pemakanan<br>*Dietary Practice*	34
H	Merokok<br>*Smoking*	35
J	Alkohol<br>*Alcohol*	44
K	Tuberkulosis<br>*Tuberculosis*	45
L	Penyakit Kusta<br>*Hansen's Disease/ Leprosy*	48
M	Denggi<br>*Dengue*	49
N	Perubatan Tradisional dan Komplementari (PT&K)<br>*Traditional and Complementary Medicine (T&CM)*	55
P	Kehilangan Upaya<br>*Disability*	110
R	Pemeriksaan Klinikal<br>*Clinical Assessment*	111
R3	Pemeriksaan Tekanan Darah<br>*Blood Pressure Measurement*	113
R4	Pemeriksaan Biokimia<br>*Biochemistry Test*	113
R5	Anemia; Pemeriksaan Hemoglobin<br>*Haemoglobin Test*	114

MODUL A2 : SOSIODEMOGRAFI / *SOCIODEMOGRAPHY*

A2: INDIVIDU BERUMUR 13 TAHUN DAN KE ATAS

Soalan untuk diisi oleh penemuramah : Bulatkan **SATU** jawapan sahaja

A2000 : Siapakah yang telah menjawab borang soal selidik ini?

1. Ahli isirumah sendiri
2. Ahli isirumah dibantu oleh penterjemah
3. Proksi (bagi pihak ahli isirumah)
4. Proksi dengan bantuan penterjemah

A2100	Nama ahli isi rumah / Name of household member : ____________	
A2104	Apakah hubungan anda dengan ... (nama ketua isirumah)?<br>*What is your relationship to ... (name of the head of household)?*	1 Ketua isirumah / *Head of Household*<br>2 Suami atau isteri / *Spouse*<br>3 Ibubapa / *Parents*<br>4 Anak / *Child*<br>5 Datuk/ Nenek atau moyang / *Grand-or great-grandparents*<br>6 Cucu atau cicit / Grand- or great-grandchild<br>7 Adik-beradik / *Siblings*<br>8 Mertua / *Parent-in-law*<br>9 Menantu / *Son- or daughter-in-law*<br>10 Ipar Duai / *Brother-or sister-in-law*<br>11 Saudara-mara lain / *Other relatives*<br>12 Kawan / *Friend*<br>13 Pekerja seperti pembantu rumah, tukang kebun, pemandu, dll. / *Workers such as live-in housemaid / gardener / driver etc.*<br>14 Lain-lain / *Others*<br><br>(-7) TT (-9) EJ
A2110	Bila tarikh lahir anda?<br>*When is your birth date?*	*D D* / *M M* / *Y Y Y Y*
**[PENEMURAMAH : Sekiranya 'TT' tuliskan '01' untuk hari '07' untuk bulan]**		
A2111	Berapa umur...?<br>*How old are...?*	☐ Tahun genap (-7) TT (-9) EJ
A2112	Apakah nombor kad pengenalan anda?<br>*What is your identification number?*	No. KP Baru / *New IC / MyKid*<br>☐<br>No. Passport / *Passport No.*<br>☐<br>No. Kad Pengenalan yang lain Tentera-Polis-Sijil lahir-Lain-lain / *Army-Police-Birth cert-Others*<br>☐<br><br>**[PERINGATAN : Pastikan nombor pengenalan BETUL]**

2 Borang Soal Selidik Individu Responden 13 Tahun Dan Ke Atas / *Individual Questionnaire Respondent 13 Years Old and Above*

A2120	Apakah bangsa anda? *What is your ethnicity?*	Kod [ ]<br><br>Lain-lain, nyatakan : ____________<br><br>(-7) TT (-9) EJ<br><br>**[PENEMURAMAH : Rujuk Kod B]**
A2130	Apakah taraf **kewarganegaraan** anda?<br>*What is your **citizenship** status?*	1. Warganegara Malaysia / *Malaysian Citizen*<br>2. Permastautin tetap / *Permanent Resident of Malaysia*<br>3. Bukan warganegara Malaysia / *Non-Malaysian Citizen*<br><br>(-7) TT (-9) EJ
A2140	Apakah **taraf perkahwinan** anda?<br>*What is your **marital status**?*	1. Tidak pernah berkahwin / *Never married*<br>2. Berkahwin / *Married*<br>3. Berpisah / *Separated*<br>4. Janda / Duda / *Divorcee*<br>5. Balu / *Widow / er*<br>6. Tinggal bersama pasangan / *Cohabiting*<br>7. Lain-lain contohnya lelaki / perempuan simpanan *Others eg. Mistress / masterers*<br><br>(-7) TT (-9) EJ
A2200	Apakah tahap pendidikan **tertinggi** anda?<br>*What is your **highest** education level?*	1. Tidak pernah bersekolah / *Never been to school*<br>2. Tidak habis sekolah rendah / *Did not complete primary school*<br>3. Tamat darjah 6 / *Completed standard 6*<br>4 Tamat tingkatan 3 / *Completed form 3*<br>5. Tamat tingkatan 5 / *Completed form 5*<br>6. Tamat tingkatan 6 / sijil/ diploma / *Completed form 6 / certificate / diploma*<br>7. Tamat pengajian peringkat sarjana muda / *Completed Bachelors degree*<br>8. Tamat pengajian peringkat sarjana / *Completed Masters degree*<br>9. Tamat pengajian peringkat kedoktoran (PHD) / *Completed Doctoral qualification (PhD)*<br>10. Lain-Lain / *Others*<br><br>(-7) TT (-9) EJ
A2210	Adakah anda bekerja? *Are you working?*	1. Ya / *Yes*..... **Ke A2213**<br>2. Tidak / *No*<br><br>(-7) TT (-9) EJ

A2211	Adakah anda bekerja dalam satu bulan yang lepas?<br>*Did you work in the last one month?*<br><br>**[PROBE : tanyakan samada menerima bayaran]**	1. Ya, dengan bayaran / *Yes, with payment...* **ke A2213**<br>2. Ya, tanpa bayaran / *Yes, without payment...* **ke A2213**<br>3. Tidak / *No*<br><br>(-7) TT (-9) EJ
A2212	Apa yang anda lakukan dalam satu bulan yang lepas?<br>*What did you mainly do in the last one month?*<br><br>Pilih **satu** jawapan sahaja /<br>*Choose only **one** answer*	1. Mempunyai pekerjaan tapi tidak bekerja / *Have a job but happened not to work*<br>2. Tidak bekerja, sedang mencari kerja / *Unemployed, looking for work*<br>3. Masih belajar, universiti / *Still schooling, university*<br>4. Menjaga rumah dan anak-anak / *Homemaker, care of children*<br>5. Sakit kronik, kurang upaya / *Chronically ill, handicapped*<br>6. Menjaga pesakit kronik atau orang kurang upaya / *Care of chronically ill, handicapped, elderly*<br>7. Bersara / *Retired*<br>8. Sakit, dengan sijil perubatan / *Sick, with a medical certificate*<br>9. Tua / *Old age*<br>10. Lain-lain / *Others*<br><br>(-7) TT (-9) EJ
**[PENEMURAMAH : Soalan Lompat-Terus ke A2220 Selepas soalan ini]**		
A2213	Adakah anda...<br>*Are you a...*	1. Pekerja Kerajaan / Government Employee<br>2. Pekerja Badan Berkanun / *Semi Government Employee*<br>3. Pekerja Swasta / *Private Employee*<br>4. Pekerja Sendiri / *Self Employed*<br>5. Pekerja tidak diupah bayar / *Unpaid worker*<br><br>(-7) TT (-9) EJ
A2213s	Adakah anda bekerja lebih daripada satu sektor, nyatakan kod sektor...<br>*Are you working in more than one sector, specify sector's code...*<br><br>**A2213 ditanya jika :**<br>**A2210 = 'YA' atau A2211 = 'YA'**	**[PENEMURAMAH : Bacakan pilihan jawapan]**<br>1. Ya / Yes<br>Sila nyatakan sektor-sektor anda bekerja<br>____________________<br>*Please specify sectors you are working in*<br>____________________<br><br>2. Tidak, bekerja dalam satu sektor sahaja<br>No, works in only one sector<br><br>**Dengan merujuk kepada kod jawapan soalan A2213. Contohnya 1+3 merujuk kepada pekerja sektor kerajaan dan juga sektor swasta**

4 Borang Soal Selidik Individu Responden 13 Tahun Dan Ke Atas /
Individual Questionnaire Respondent 13 Years Old and Above

BAGI SOALAN A2214,A2215,A2216 & A2217 HANYA UNTUK AHLI ISIRUMAH BERUMUR 15 TAHUN DAN KE ATAS. JIKA BERUMUR KURANG DARI 15, TERUS KE A2220<br>*QUESTION A2214,A2215,A2216 & A2217 ARE ONLY FOR MEMBER OF THE HOUSEHOLD AGED 15 YEARS AND ABOVE. IF AGE BELOW 15, GO TO A2220*		
A2214	Apakah pekerjaan utama anda?<br>*What is your main job title?*	Nyatakan dan pilih salah satu kategori pekerjaan di bawah<br>*Please specify and choose only one of below job category*<br><br>1. Pengurus dan Pegawai Atasan / *Managers and Senior Officials*<br>2. Profesional / *Professionals*<br>3. Juruteknik dan Profesional Bersekutu / *Technician and Associate Professionals*<br>4. Pekerja Perkeranian / *Clerical Workers*<br>5. Pekerja Perkhidmatan dan Jualan / *Services & Sales Workers*<br>6. Pekerja Berkemahiran / *Skilled Workers*<br>7. Pekerja Tidak Berkemahiran atau Pekerja Am / *Unskilled or General Workers*<br>8. Angkatan Tentera / *Armed Forces*<br><br>(-7) TT (-9) EJ
A2215	Apakah bidang pekerjaan anda?<br>*In which field are you working?*	Sila pilih hanya SATU bidang dibawah<br>*Please choose only ONE field from below*<br><br>1. Pembuatan / *Manufacturing*<br>2. Perdagangan Borong dan Runcit; Pembaikan Kenderaan Bermotor dan Motosikal / *Wholesale and Retail Trade; Repair of Motor Vehicle and Motorcycles*<br>3. Pertanian, Penternakan, Perhutanan dan Perikanan / *Agriculture, Live Stock Farming, Forestry and Fishing*<br>4. Pembinaan / *Construction*<br>5. Penginapan; Aktiviti Perkhidmatan Makanan dan Minuman / *Accommodation; Food And Beverage Service Activities*<br>6. Pendidikan / *Education*<br>7. Pentadbiran Awam dan Pertahanan; Aktiviti Keselamatan Sosial Wajib / *Public Administration and Defence; Compulsory Social Security*<br>8. Pentadbiran (Bukan Awam) dan Perkhidmatan Sokongan / *Administrative (Private Sector) and Support Service Activities*<br>9. Pengangkutan dan Penyimpanan / *Transportation and Storage*<br>10. Aktiviti Kesihatan Kemanusiaan dan Kerja Sosial / *Human Health and Social Work Activities*<br>11. Aktiviti Kewangan dan Insurans / Takaful / *Financial and Insurance / Takaful Activities*<br>12. Aktiviti Perundingan Profesional, Saintifik dan Teknik / *Professional Consultation, Scientific and Technical Activities*<br>13. Lain-lain / *Others*<br><br>(-7) TT (-9) EJ

A2216	Nyatakan tempoh pekerjaan anda sekarang.<br>*State the duration of your current work.*<br>[ ] tahun / *year* [ ] bulan / *month*<br>**Menggenapkan ke bulan yang terdekat jika kurang dari setahun; ke tahun yang terdekat jika lebih dari setahun**	
A2217	Apakah jenis sistem waktu bekerja anda?<br>*What is your working time system?*	1. Waktu bekerja tetap (Kurang dari 48 jam seminggu) / *Regular working time (Less than 48 hours per week)*<br>2. Bekerja lebih masa (Melebihi 48 jam seminggu) / *Extended Hours (More than 48 hours per week)*<br>3. Hari Bekerja tidak tetap / *Stanggered Days*<br>4. Bekerja shif / *Shift hours*<br>5. Bekerja Perjalanan Jauh / *Long haul*<br>6. Pekerja Sambilan / *Part-time*<br>(-7) TT (-9) EJ
Berapakah purata pendapatan kasar anda sebulan, dari segi...<br>*What is your average personal gross monthly income, in terms of...*		**Sekiranya TIADA pendapatan tuliskan '0'**
A2220	Pendapatan dari bekerja / gaji / upah / pencen<br>*Income from work / salary / pension*<br>(-7) TT (-9) EJ	**RM** [ ] Sebulan / *Monthly*
A2221	Wang yang diterima daripada ahli isirumah lain<br>*Money received from other household members*<br>(-7) TT (-9) EJ	**RM** [ ] Sebulan / *Monthly*
A2222	Wang dari sumber lain, cth daripada kutipan sewa aset, wang daripada ahli keluarga di luar isi rumah, biasiswa, kebajikan masyarakat, Baitulmal dll<br>*Money from other source e.g from rented assets collection, from family member outside the household, scholarship, welfare, Baitulmal etc*<br>(-7) TT (-9) EJ	**RM** [ ] Sebulan / *Monthly*

MODUL C: KENCING MANIS / *DIABETES MELLITUS*

**KRITERIA KELAYAKAN: AHLI ISIRUMAH BERUMUR 18 TAHUN DAN KE ATAS**<br>***MEMBER OF THE HOUSEHOLD AGED 18 YEARS AND ABOVE***			
Bulatkan jawapan yang bersesuaian / *Please circle the appropriate answer.*			
Sekarang saya ingin bertanya mengenai kesihatan anda, terutamanya berkenaan kencing manis<br>*Now I would like to ask you about your health, particularly in regards to diabetes.*			
C010	Dalam tempoh 12 bulan yang lepas, pernahkah anda menjalani pemeriksaan paras gula dalam darah?<br>*Have you ever had your blood sugar measured in the past 12 months?*<br><br>1. Ya / *Yes*<br>2. Tidak / *No*<br><br>(-7) TT (-9) EJ	C020	Pernahkah anda diberitahu oleh doktor ataupun Penolong Pegawai Perubatan (PPP) bahawa anda menghidap penyakit kencing manis atau diabetes?<br>*Have you ever been told by a doctor or Assistant Medical Officer that you have diabetes?*<br><br>1. Ya / *Yes*<br>2. Tidak / *No*<br><br>(-7) TT (-9) EJ<br><br>**Jika "Ya" terus ke C031-C035, dan C040, jika "Tidak", "TT" atau "EJ" terus ke Modul D**
Apakah jenis rawatan atau nasihat yang anda terima daripada doktor (atau anggota kesihatan lain) untuk mengawal penyakit kencing manis?<br>*What kind of treatments or advice for diabetes are you currently receiving from doctor or other health worker?*			
C031	Insulin / *Insulin*<br><br>1. Ya / *Yes*<br>2. Tidak / *No*<br><br>(-7) TT (-9) EJ	C032	Ubat-ubatan dalam masa 2 minggu lepas<br>*Drugs (medication) in the past two weeks*<br><br>1. Ya / *Yes*<br>2. Tidak / *No*<br><br>(-7) TT (-9) EJ
C033	Nasihat diet khusus untuk penyakit kencing manis / *Diabetic diet*<br><br>1. Ya / *Yes*<br>2. Tidak / *No*<br><br>(-7) TT (-9) EJ	C034	Nasihat untuk kurangkan berat badan<br>*Advice to lose weight*<br><br>1. Ya / *Yes*<br>2. Tidak / *No*<br><br>(-7) TT (-9) EJ
C035	Nasihat untuk mula bersenam atau lebihkan senaman / *Advice to start or do more exercise*<br><br>1. Ya / *Yes*<br>2. Tidak / *No*<br><br>(-7) TT (-9) EJ		

26 Borang Soal Selidik Individu Responden 13 Tahun Dan Ke Atas /
Individual Questionnaire Respondent 13 Years Old and Above

MODUL D: TEKANAN DARAH TINGGI / *HYPERTENSION*

**KRITERIA KELAYAKAN: AHLI ISIRUMAH BERUMUR 18 TAHUN DAN KE ATAS**<br>***MEMBER OF THE HOUSEHOLD AGED 18 YEARS AND ABOVE***			
Bulatkan jawapan yang bersesuaian/ *Please circle the appropriate answer.*			
Sekarang saya ingin bertanya mengenai kesihatan anda, terutamanya berkenaan tekanan darah tinggi.<br>*Now I would like to ask you about your health, particularly in regards to high blood pressure.*			
D010	Dalam tempoh 12 bulan yang lepas, pernahkah anda menjalani pemeriksaan tekanan darah?<br>*Have you ever had your blood pressured measured in the past 12 months?*<br><br>1. Ya / *Yes*<br>2. Tidak / *No*<br><br>(-7) TT (-9) EJ	D020	Pernahkah anda diberitahu oleh doktor ataupun Penolong Pegawai Perubatan (PPP) bahawa tekanan darah anda adalah tinggi?<br>*Have you ever been told by a doctor or Assistant Medical Officer that you have raised blood pressure or hypertension?*<br><br>1. Ya / *Yes*<br>2. Tidak / *No*<br><br>(-7) TT (-9) EJ<br><br>**Jika "Ya" terus ke D031-D034, dan D040, jika "Tidak", "TT" atau "EJ" terus ke Modul E/R3**
Apakah jenis rawatan atau nasihat yang anda terima daripada doktor (atau anggota kesihatan lain) untuk mengawal penyakit darah tinggi? / *What kind of treatments or advice for high blood pressure or hypertension are you currently receiving from doctor or other health worker?*			
D031	Ubat-ubatan sejak 2 minggu lepas<br>*Drugs (medication) in the past two weeks*<br><br>1. Ya / *Yes*<br>2. Tidak / *No*<br><br>(-7) TT (-9) EJ	D032	Nasihat untuk kurangkan garam dalam makanan / *Advice to reduce salt intake*<br><br>1. Ya / *Yes*<br>2. Tidak / *No*<br><br>(-7) TT (-9) EJ
D033	Nasihat untuk kurangkan berat badan<br>*Advice to lose weight*<br><br>1. Ya / *Yes*<br>2. Tidak / *No*<br><br>(-7) TT (-9) EJ	D034	Nasihat untuk mula bersenam atau lebihkan senaman / *Advice to start or do more exercise*<br><br>1. Ya / *Yes*<br>2. Tidak / *No*<br><br>(-7) TT (-9) EJ

D040	Di manakah anda selalunya mendapat rawatan untuk penyakit darah tinggi?<br>*Where do you usually seek treatment for your high blood pressure?*<br><br>1 Klinik kerajaan/ *Government clinic*<br>2 Klinik swasta / *Private clinic*<br>3 Hospital kerajaan/ *Government hospital*<br>4 Hospital swasta / *Private hospital*<br>5 Farmasi (rawatan sendiri) / *Pharmacy (self-medicating) medicating*<br>6 Pengamal rawatan tradisional, herba atau komplementari /<br>*Traditional, herbal and complementary medicine*<br><br>1. Ya / *Yes*<br>2. Tidak / *No*<br><br>(-7) TT (-9) EJ
D041	Pernakah anda mengalami serangan jantung ataupun sakit dada berpunca dari penyakit jantung (angina) atau angin ahmar?<br>*Have you ever had a heart attack or chest pain from heart disease (angina) or a stroke (cerebrovascular accident)?*<br><br>1. Ya / *Yes*<br>2. Tidak / *No*<br><br>(-7) TT (-9) EJ

MODUL E : PARAS KOLESTEROL TINGGI / *HYPERCHOLESTEROLAEMIA*

KRITERIA KELAYAKAN : AHLI ISIRUMAH BERUMUR 18 TAHUN DAN KE ATAS
MEMBER OF THE HOUSEHOLD AGED 18 YEARS AND ABOVE

Bulatkan jawapan yang bersesuaian/ *Please circle the appropriate answer.*

Sekarang saya ingin bertanya mengenai kesihatan anda, terutamanya berkenaan paras kolesterol yang tinggi.
Now I would like to ask you about your health, particularly in regards to high cholesterol.

E010	Dalam tempoh 12 bulan yang lepas, pernahkah anda menjalani pemeriksaan paras kolesterol dalam darah?<br>*Have you ever had your total blood cholesterol measured in the past 12 months?*<br><br>1. Ya / *Yes*<br>2. Tidak / *No*<br><br>(-7) TT (-9) EJ	E020	Pernahkah anda diberitahu oleh doktor ataupun Penolong Pegawai Perubatan (PPP) bahawa paras kolesterol darah anda adalah tinggi?<br>*Have you ever been told by a doctor or Assistant Medical Officer that you have high cholesterol?*<br><br>1. Ya / *Yes*<br>2. Tidak / *No*<br><br>(-7) TT (-9) EJ<br><br>**Jika "Ya" terus ke E031-E034, dan E040, jika "Tidak", "TT" atau "EJ" terus ke Modul F**

Apakah jenis rawatan atau nasihat yang anda terima daripada doktor (atau anggota kesihatan lain) untuk mengawal paras kolesterol tinggi? / *What kind of treatments or advice for high cholesterol are you currently receiving from doctor or other health worker?*

E031	Ubat-ubatan sejak 2 minggu lepas<br>*Drugs (medication) in the past two weeks*<br><br>1. Ya / *Yes*<br>2. Tidak / *No*<br><br>(-7) TT (-9) EJ	E032	Nasihat diet khusus seperti rendah lemak atau rendah kolesterol<br>*Special low fat or low cholesterol diet*<br><br>1. Ya / *Yes*<br>2. Tidak / *No*<br><br>(-7) TT (-9) EJ
E033	Nasihat untuk kurangkan berat badan<br>*Advice to lose weight*<br><br>1. Ya / *Yes*<br>2. Tidak / *No*<br><br>(-7) TT (-9) EJ	E034	Nasihat untuk mula bersenam atau lebihkan senaman<br>*Advice to start or do more exercise*<br><br>1. Ya / *Yes*<br>2. Tidak / *No*<br><br>(-7) TT (-9) EJ

E040	Di manakah anda selalunya mendapat rawatan untuk penyakit darah tinggi?<br>*Where do you usually seek treatment for your high blood pressure?*<br><br>1 Klinik kerajaan/ *Government clinic*<br>2 Klinik swasta / *Private clinic*<br>3 Hospital kerajaan/ *Government hospital*<br>4 Hospital swasta / *Private hospital*<br>5 Farmasi (rawatan sendiri) / *Pharmacy (self-medicating) medicating*<br>6 Pengamal rawatan tradisional, herba atau komplementari<br>*Traditional, herbal and complementary medicine*<br><br>(-7) TT (-9) EJ

MODUL F : AKTIVITI FIZIKAL / *PHYSICAL ACTIVITY*

**KRITERIA KELAYAKAN: AHLI ISIRUMAH BERUMUR 16 TAHUN DAN KE ATAS**<br>***MEMBER OF THE HOUSEHOLD AGED 16 YEARS AND ABOVE***			
Bulatkan jawapan yang bersesuaian/ *Please circle the appropriate answer.*			
**FIKIRKAN TENTANG AKTIVITI FIZIKAL LASAK YANG ANDA LAKUKAN.**<br>***PLEASE NOTE THE VIGOROUS PHYSICAL ACTIVITY THAT YOU DO.***			
F010	Dalam tempoh 7 hari yang lepas, berapa harikah anda telah melakukan aktiviti fizikal lasak (contohnya mengangkat barang berat, mencangkul, senaman aerobik atau berbasikal laju dan lain-lain) sekurang-kurangnya 10 minit pada suatu masa ?<br>*In the past 7 days, how many days have you done vigorous physical activity (eg: carry heavy weights, till the earth, aerobic exercises or fast cycling and others) for at least 10 minutes per session?*<br>☐ hari seminggu<br>(-8) Tiada aktiviti berat<br>(-7) TT (-9) EJ	F011	Pada hari anda yang melakukan aktiviti fizikal lasak, berapa lamakah anda melakukannya?<br>*On the day you carry out the vigorous physical activity, how long do you do this activity?*<br>☐ minit<br>(-7) TT (-9) EJ
**FIKIRKAN TENTANG AKTIVITI FIZIKAL SEDERHANA YANG ANDA LAKUKAN.**<br>***PLEASE NOTE THE MODERATE PHYSICAL ACTIVITY THAT YOU DO.***			
F020	Dalam tempoh 7 hari yang lepas, berapa harikah anda telah melakukan aktiviti fizikal sederhana (contohnya mengangkat muatan ringan, mengelap lantai, berbasikal pada kelajuan biasa dan lain-lain) sekurang-kurangnya 10 minit pada suatu masa tidak termasuk berjalan kaki?<br>*In the past 7 days, how many days have you done moderate physical activity (eg: carry light weights, mop the floor, or normal rate of cycling and others) for at least 10 minutes per session? This does not include walking*<br>☐ hari seminggu<br>(-8) Tiada aktiviti berat<br>(-7) TT (-9) EJ	F021	Pada hari anda yang melakukan aktiviti fizikal sederhana, berapa lamakah anda melakukannya?<br>*On the day you carry out the moderate physical activity, how long do you do this activity?*<br>☐ minit<br>(-7) TT (-9) EJ

32 Borang Soal Selidik Individu Responden 13 Tahun Dan Ke Atas / *Individual Questionnaire Respondent 13 Years Old and Above*

**FIKIRKAN TENTANG AKTIVITI BERJALAN KAKI YANG ANDA LAKUKAN.**<br>***PLEASE NOTE THE AMOUNT OF WALKING THAT YOU DO.***			
F030	Dalam tempoh 7 hari yang lepas, berapa harikah anda telah berjalan kaki selama sekurang-kurangnya 10 minit pada sesuatu masa?<br>*In the past 7 days, how many days have you walked for at least 10 minutes per session?*<br>[ ] hari seminggu<br>(-8) Tiada aktiviti berjalan kaki<br>(-7) TT (-9) EJ	F031	Pada salah satu daripada hari berkenaan, berapakah masa yang anda gunakan untuk berjalan kaki?<br>*On one of these days that you walked, how long do you spend walking?"*<br>[ ] minit<br>(-7) TT (-9) EJ
**FIKIRKAN TENTANG AKTIVITI BARING DAN DUDUK YANG ANDA LAKUKAN.**<br>***PLEASE NOTE THE SEDENTARY ACTIVITY THAT YOU DO.***			
F040	Biasanya dalam sehari, berapa jamkah yang anda gunakan untuk duduk atau berbaring termasuk di tempat kerja, di rumah, di waktu lapang dan semasa perjalanan, TETAPI TIDAK TERMASUK waktu tidur?<br>*Normally in a day, how many hours do you spend on sitting or lying down including the workplace, in the house, in your free time and while travelling. BUT NOT INCLUDING the time spent sleeping?*<br>[ ] jam<br>(7) TT (9) EJ		

MODUL G : AMALAN PEMAKANAN / *DIETARY PRACTICE*

KRITERIA KELAYAKAN: AHLI ISIRUMAH BERUMUR 18 TAHUN DAN KE ATAS
MEMBER OF THE HOUSEHOLD AGED 18 YEARS AND ABOVE

Bulatkan jawapan yang bersesuaian/ *Please circle the appropriate answer.*

G010	Kebiasaannya, dalam seminggu berapa hari anda makan buah-buahan?<br>*In a typical week, how many days do you eat fruits?"*<br><br>[ ] Bil Hari<br><br>(-7) TT (-9) EJ	G011	Biasanya, pada hari yang anda makan buah buahan, berapa banyak hidangan anda makan dalam sehari?<br>*Usually on the day that you eat fruits, how much did you eat in a day?*<br><br>[ ] Bil Hidangan<br><br>(-7) TT (-9) EJ<br><br>**Rujuk kad contoh satu hidangan makanan**<br>***Refer Examples of One Food Serving***
G020	Kebiasaannya, dalam seminggu berapa hari anda makan sayur-sayuran/ulam-ulaman?<br>*In a typical week, how many days do you eat vegetables / ulam-ulaman?*<br><br>[ ] Bil Hari<br><br>(-7) TT (-9) EJ	G021	Biasanya, pada hari yang anda makan sayur-sayuran/ulam-ulaman, berapa banyak anda makan dalam sehari?<br>*Usually on the day that you eat vegetables /ulam-ulaman, how much did you eat in a day?"*<br><br>[ ] Bil Hidangan<br><br>(-7) TT (-9) EJ<br><br>**Rujuk kad contoh satu hidangan makanan**<br>***Refer Examples of One Food Serving***
G030	Kebiasaannya, berapa gelas anda minum air kosong dalam sehari?<br>Typically, how many glasses of plain water you drink in a day?<br><br>[ ] Bil Gelas<br><br>(-7) TT (-9) EJ<br><br>**Rujuk kad contoh satu hidangan makanan / *Refer Examples of One Food Serving***		

34 Borang Soal Selidik Individu Responden 13 Tahun Dan Ke Atas /
Individual Questionnaire Respondent 13 Years Old and Above

MODUL H : MEROKOK / *SMOKING*

KRITERIA KELAYAKAN UNTUK MENJAWAB: 15 TAHUN DAN KEATAS<br>*ELIGIBILITY CRITERIA: 15 YEARS AND ABOVE*			
H1	**Status Merokok Terkini**<br>***Current Tobacco Smoking Status***<br><br>Pada ketika ini adakah anda menghisap tembakau setiap hari, kurang daripada setiap sehari, atau tidak sama sekali?<br>*Do you currently smoke tobacco on a daily basis, less than daily, or not at all?*<br><br>1. Setiap Hari / *Daily* - **ke soalan H3**<br>2. Kurang Dari Setiap Hari<br>*Less Than Daily* - **ke soalan H2a**<br>3. Tidak Sama Sekali / *Not At All* - **ke soalan H2b**<br><br>(-7) TT (-9) EJ<br><br>**Jika TT atau EJ ke Soalan H3**	H2a	**Status Merokok Setiap Hari Terdahulu**<br>***Past Daily Smoking Status***<br><br>Pernahkah anda menghisap tembakau setiap hari pada masa lalu?<br>*Have you smoked tobacco daily in the past?*<br><br>1. Ya / *Yes* - **ke Soalan H3**<br>2. Tidak / *No* - **Ke soalan H3**<br><br>(-7) TT (-9) EJ<br><br>**Jika TT atau EJ ke Soalan H3**
H2b	**Status Merokok Terdahulu / *Past Smoking Status***<br><br>Pada masa lalu, pernahkah anda menghisap tembakau setiap hari, kurang daripada setiap sehari, atau tidak sama sekali? / *In the past, have you smoked tobacco on a daily basis, less than daily, or not at all?*<br><br>JIKA RESPONDEN TELAH MENJAWAB KEDUA-DUANYA "SETIAP HARI" DAN "KURANG DARIPADA SETIAP SEHARI" PADA MASA LALU, TANDAKAN "SETIAP HARI"<br>*IF RESPONDENT HAS DONE BOTH "DAILY" AND "LESS THAN DAILY" IN THE PAST, CHECK "DAILY"*<br><br>1. Setiap Hari / *Daily*<br>2. Kurang Dari Setiap Hari<br>*Less Than Daily*<br>3. Tidak Sama Sekali / *Not At All* – **ke soalan H4**<br><br>(-7) TT (-9) EJ<br><br>**Jika menjawab (3)Tidak sama sekali, (-7) TT, atau (-9) EJ terus ke soalan H4**		

R2021	Tarikh Pengukuran Antropometri / *Anthropometric Measurement Date:*<br>[ ][ ] Hari / *Day* [ ][ ] Bulan / *Month* [ ][ ][ ][ ] Tahun / *Year*
	**Berat Badan / *Body Weight***
R2031	Ukuran 1/ *1st Measurement* [ ][ ][ ] . [ ][ ] kg
R2032	Ukuran 2/ *2nd Measurement* [ ][ ][ ] . [ ][ ] kg
	(-6) Tidak berkaitan/ *Not applicable* (-9) Enggan diukur/ *Refuse to be measured*
	**Tinggi / *Height***
R2041	Ukuran 1/ *1st Measurement* [ ][ ][ ] . [ ][ ] cm
R2042	Ukuran 2/ *2nd Measurement* [ ][ ][ ] . [ ][ ] cm
	(-6) Tidak berkaitan/ *Not applicable* (-9) Enggan diukur/ *Refuse to be measured*
	**Setengah depa lengan/ *Half-arm span***
R2051	Ukuran 1/ *1st Measurement* [ ][ ][ ] . [ ][ ] cm
R2052	Ukuran 2/ *2nd Measurement* [ ][ ][ ] . [ ][ ] cm
	(6) Tidak berkaitan/ *Not applicable* (9) Enggan diukur/ *Refuse to be measured*
	**Ukur lilit pinggang / *Waist circumference***
R2051	Ukuran 1/ *1st Measurement* [ ][ ][ ] . [ ][ ] cm
R2052	Ukuran 2/ *2nd Measurement* [ ][ ][ ] . [ ][ ] cm
	(-6) Tidak berkaitan/ *Not applicable* (-9) Enggan diukur/ *Refuse to be measured*
	**Ukur lilit betis/ *Calf circumference***<br>**KRITERIA KELAYAKAN: RESPONDEN BERUSIA 60 TAHUN DAN KEATAS**<br>***ELIGIBILITY CRITERIA: RESPONDENTS AGED 60 YEARS AND ABOVE***
R2051	Ukuran 1/ *1st Measurement* [ ][ ][ ] . [ ][ ] cm
R2052	Ukuran 2/ *2nd Measurement* [ ][ ][ ] . [ ][ ] cm
	(-6) Tidak berkaitan/ *Not applicable* (-9) Enggan diukur/ *Refuse to be measured*

ID : NEGERI | DP | DB | BP | STRATA | TK | ISI RUMAH | INDIVIDU

MODUL R3 : PEMERIKSAAN TEKANAN DARAH
BLOOD PRESSURE MEASUREMENT

KRITERIA KELAYAKAN RESPONDEN: BERUSIA 18 TAHUN DAN LEBIH			
Bacaan tekanan darah / *Blood pressure reading*			
R3010	Sistolik / *Systolic*	1 Bacaan pertama / *First reading*	☐☐☐
R3020	Diastolik / *Diastolic*	1 Bacaan pertama / *First reading*	☐☐☐
R3030	Sistolik / *Systolic*	2 Bacaan kedua / *Second reading*	☐☐☐
R3040	Diastolik / *Diastolic*	2 Bacaan kedua / *Second reading*	☐☐☐
R3050	Sistolik / *Systolic*	3 Bacaan ketiga / *Third reading*	☐☐☐
R3060	Diastolik / *Diastolic*<br>(-9) Enggan diperiksa / *Refuse to be examined*	3 Bacaan ketiga / *Third reading*	☐☐☐

** **Bagi Modul Berikutnya, jika wanita mengandung hanya perlu lakukan Modul R5 sahaja. Jika tidak mengandung, perlu lakukan Modul R4 & R5.**

MODUL R4 : PEMERIKSAAN BIOKIMIA / *BIOCHEMISTRY TEST*

KRITERIA KELAYAKAN RESPONDEN : BERUSIA 18 TAHUN DAN LEBIH **SERTA TIDAK MENGANDUNG**		
R4010	Dalam tempoh 8 jam yang lepas, adakah anda makan dan minum selain dari air kosong?<br>*During the past 8 hours have you had anything to eat or drink, other than water?*	1. Ya / *Yes*<br>2. Tidak / *No*<br>(-7) TT (-9) EJ
R4020	Paras glukosa kapilari<br>*Capillary blood glucose level*	1 ☐☐ . ☐ mmol/L<br>(-9) Enggan diperiksa / *Refuse to be examined*
R4030	Bacaan paras kolesterol<br>*Blood cholesterol level measurement*	1 ☐☐ . ☐ mmol/L<br>(-9) Enggan diperiksa / *Refuse to be examined*

MODUL J : ALKOHOL / *ALCOHOL*

ID

Negeri	DP	DB	BP	STRATA	TK	ISI RUMAH	INDIVIDU

Sila tandakan (√) pada kotak yang bersesuaian. *Please (√) in the appropriate box.*

1. Apakah jenis minuman berakohol/arak/minuman keras yang paling kerap anda minum?<br>*What type of alcoholic beverages did you most frequently consume?*	Sila tandakan (√) sekali sahaja iaitu pada minuman yang paling kerap diminum.<br>*Please tick (√) once only indicating the alcoholic beverages that is often consumed.*<br>☐ Shandy<br>*Shandy*<br>☐ Bir ATAU Lager ATAU Ale ATAU Stout<br>*Beer OR Lager OR Ale OR Stout*<br>☐ Wain ATAU Cider ATAU Champagne ATAU Peri ATAU Todi<br>*Wine Or Cider OR Champagne OR Peri Or Toddy*<br>☐ Tuak ATAU Tuak Kelapa ATAU Bahar ATAU Lihing ATAU Ijok<br>*Tuak OR Tuak Kelapa OR Bahar OR Lihing Or Ijok*<br>☐ Brandi ATAU Rum ATAU Wiski ATAU Vodka ATAU Gin ATAU Samsu ATAU Sam Cheng<br>*Brandy OR Rum OR Whisky OR Vodka OR Gin Or Samsu Or Sam Cheng*<br>☐ Montoku ATAU Langkau<br>*Montoku OR Langkau*<br>☐ Lain-lain<br>*Others*
2. Dalam tempoh 12 bulan yang lepas berapa kerapkah anda minum minuman berakohol/arak/minuman keras?<br>*For the past 12 months how often have you had a drink containing alcohol?*	☐ Tidak Pernah<br>*Never*<br>☐ Sekali sebulan atau kurang<br>*Once a month or less*<br>☐ 2-4 kali sebulan<br>*2 to 4 times a month*<br>☐ 2-3 kali seminggu<br>*2 to 3 times a week*<br>☐ 4 kali atau lebih seminggu<br>*4 or more times a week*
3. **Kebiasaannya** pada hari yang anda minum, berapa banyakkah anda minum minuman berakohol/arak/ minuman keras ?<br>Jumlah pengambilan minuman beralkohol mestilah mengikut minuman alkohol seperti yang ditunjukkan dalam Kad AA.<br>*How many alcoholic beverages do you have on a typical day when you are drinking?*<br>*The total amount of alcohol consumed should be calculated in terms of standard drink consumed per day as shown in Card AA.*	Sila rujuk Kad AA / *Please refer to Card AA*<br>☐ 1 atau 2<br>*1 or 2*<br>☐ 3 atau 4<br>*3 or 4*<br>☐ 5 atau 6<br>*5 or 6*<br>☐ 7, 8 atau 9<br>*7, 8 or 9*<br>☐ 10 atau lebih<br>*10 or more*
4. Berapa kerap anda minum enam atau lebih minuman beralkohol pada satu masa?<br>*How often do you have six or more drinks on one occasion?*	☐ Tidak Pernah<br>*Never*<br>☐ Kurang dari sekali sebulan<br>*Less than once a month*<br>☐ Sekali sebulan<br>*Monthly*<br>☐ Sekali seminggu<br>*Weekly*<br>☐ Setiap hari atau hampir setiap hari<br>*Daily or almost daily*

JIKA SOALAN 3 DI JAWAB SEBAGAI "**1 ATAU 2**" DAN SOALAN 4 DIJAWAB SEBAGAI '**TIDAK PERNAH**' TERUS KE SOALAN 10

*IF ANSWERED "**1 OR 2**" FOR QUESTION 3 AND '**NEVER**' TO QUESTIONS 4, PROCEED TO QUESTION 10*

Page 2 of 5

MODUL J : ALKOHOL / *ALCOHOL*

5. Dalam tempoh 12 bulan yang lepas, berapa kerapkah anda tidak boleh berhenti minum apabila anda mula minum minuman berakohol/arak/minuman keras?<br>*How often during the last 12 months have you found that you were not able to stop drinking once you had started?*	☐ Tidak Pernah<br>*Never*<br>☐ Kurang dari sekali sebulan<br>*Less than once a month*<br>☐ Sekali sebulan<br>*Monthly*<br>☐ Sekali seminggu<br>*Weekly*<br>☐ Setiap hari atau hampir setiap hari<br>*Daily or almost daily*
6. Dalam tempoh 12 bulan yang lepas, akibat dari minum minuman berakohol/arak/minuman keras berapa kerapkah anda tidak boleh melakukan apa yang biasanya anda lakukan?<br>*How often during the last 12 months have you failed to do what was normally expected from you because of drinking?*	☐ Tidak Pernah<br>*Never*<br>☐ Kurang dari sekali sebulan<br>*Less than once a month*<br>☐ Sekali sebulan<br>*Monthly*<br>☐ Sekali seminggu<br>*Weekly*<br>☐ Setiap hari atau hampir setiap hari<br>*Daily or almost daily*
7. Dalam tempoh 12 bulan yang lepas, selepas sesi meminum minuman berakohol/arak/minuman keras ldalam jumlah melebihi dari biasa, berapa kerapkah pada pagi esoknya anda perlu meminum minuman berakohol/arak/ minuman keras sebelum memulakan hari anda?<br>*How often during the last 12 months you needed a first drink in the morning to get yourself going after a heavy drinking session?*	☐ Tidak Pernah<br>*Never*<br>☐ Kurang dari sekali sebulan<br>*Less than once a month*<br>☐ Sekali sebulan<br>*Monthly*<br>☐ Sekali seminggu<br>*Weekly*<br>☐ Setiap hari atau hampir setiap hari<br>*Daily or almost daily*
8. Dalam tempoh 12 bulan yang lepas, berapa kerapkah anda rasa bersalah atau menyesal selepas minum minuman berakohol/arak/minuman keras?<br>*How often during the last 12 months have you had a feeling of guilt or remorse after drinking?*	☐ Tidak Pernah<br>*Never*<br>☐ Kurang dari sekali sebulan<br>*Less than once a month*<br>☐ Sekali sebulan<br>*Monthly*<br>☐ Sekali seminggu<br>*Weekly*<br>☐ Setiap hari atau hampir setiap hari<br>*Daily or almost daily*
9. Dalam tempoh 12 bulan yang lepas, berapa kerapkah anda tidak dapat mengingati apakah yang telah berlaku malam sebelumnya disebabkan anda telah mengambil minuman berakohol/arak/minuman keras?<br>*How often during the last 12 months have you been unable to remember what happened the night before because you had been drinking?*	☐ Tidak Pernah<br>*Never*<br>☐ Kurang dari sekali sebulan<br>*Less than once a month*<br>☐ Sekali sebulan<br>*Monthly*<br>☐ Sekali seminggu<br>*Weekly*<br>☐ Setiap hari atau hampir setiap hari<br>*Daily or almost daily*

Page 3 of 5

MODUL J : ALKOHOL / *ALCOHOL*

10. Pernahkah anda atau orang lain tercedera disebabkan anda meminum minuman berakohol/arak/minuman keras?<br>*Have you or someone else been injured as a result of your drinking?*	☐ Tidak<br>*No*<br>☐ Ya, tetapi bukan dalam tempoh setahun yang lepas<br>*Yes, but not in the last year*<br>☐ Ya,dalam tempoh setahun yang lalu<br>*Yes, during the last year*
11. Pernahkah saudara atau kaw an atau doktor atau anggota kesihatan mengambil berat atau mencadangkan supaya anda mengurangkan pengambilan minuman berakohol/arak/minuman keras?<br>*Has a relative or friend or a doctor or another health worker been concerned about your drinking or suggested you cut down?*	☐ Tidak<br>*No*<br>☐ Ya, tetapi bukan dalam tempoh setahun yang lepas<br>*Yes, but not in the last year*<br>☐ Ya,dalam tempoh setahun yang lalu<br>*Yes, during the last year*
12. Adakah anda masih mengambil minuman beralkohol/arak/ minuman keras dalam masa 1 bulan yang lalu?<br>*Are you still consuming alcoholic beverages in the last one month?*	☐ Ya<br>*Yes*<br>☐ Tidak<br>*No*<br>☐ Tidak Tahu<br>*Don't Know*<br>☐ Enggan Jaw ab<br>*Refuse to answer*
13. Berapa kerap anda minum enam atau lebih minuman beralkohol pada satu masa?<br>*How often do you have six or more drinks on one occasion?*	Sila rujuk Kad AA / *Please refer to Card AA*<br>☐ Tidak Pernah<br>*Never*<br>☐ Kurang dari sekali sebulan<br>*Less than once a month*<br>☐ Sekali sebulan<br>*Monthly*<br>☐ Sekali seminggu<br>*Weekly*<br>☐ Setiap hari atau hampir setiap hari<br>*Daily or almost daily*

Page 4 of 5

MODUL J : ALKOHOL / *ALCOHOL*

KAD AA / *CARD AA*

Pilihan Jawapan / *Choice of Answer*

Gambar rajah 1 menunjukkan ukuran bagi 1 minuman alkohol (1 standard drink). Sila gunakan contoh di bawah bagi menghitung jumlah minuman yang diambil dalam sehari.
Table 1 shows equivalent of 1 standard drink. Please use the examples given in the table below to calculate the total amount of drinks consumed per typical day when you are drinking.

Gambar rajah 1:1 minuman alkohol
Table 1:1 standard drink of alcoholic beverage

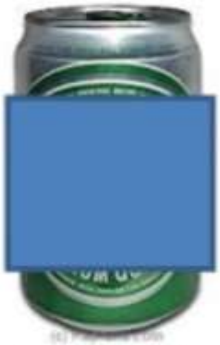 320 ml (1 tin) bir<br>*320 ml (1 tin) of beer*<br><br>Kandungan alkohol >2% & <10%<br>*Alcohol content >2% & <10%*	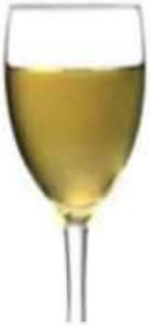 140 ml wain<br>*140 ml of wine*<br><br>Kandungan alkohol 7% ke 15%<br>*Alcohol content 7% to 15%*	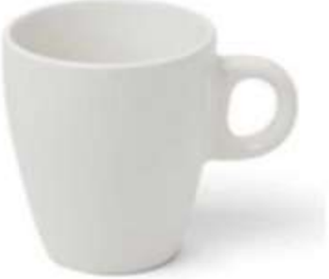 150 ml todi / bahar<br>*150 ml of toddy / bahar*<br><br>Kandungan alkohol 7% ke 15%<br>*Alcohol content 7% to 15%*
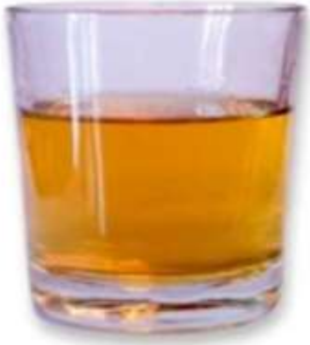 100 ml tuak / lihing<br>*100ml of tuak / lihing*<br><br>Kandungan alkohol 7% ke 15%<br>*Alcohol content 7% to 15%*	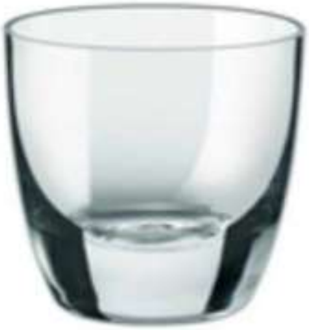 80 ml montoku<br>*80 ml of montoku*<br><br>Kandungan alkohol >30%<br>*Alcohol content > 30%*	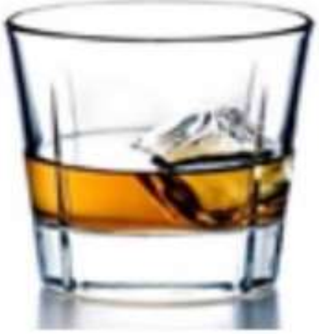 30ml langkau/samsu/brandi/wiski<br>*30ml of langkau/samsu/brandy/ whisky*<br><br>Kandungan alkohol >30%<br>*Alcohol content > 30%*

Page 5 of 5

Contoh Satu Hidangan Buah-buahan dan Sayur-sayuran

Jenis Makanan	1 Hidangan	Contoh
**A. BUAH-BUAHAN**		
Epal, pisang, limau manis, mangga, pir cina	1 biji sederhana	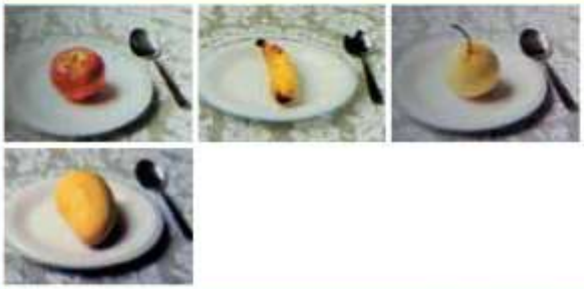
Betik, tembikai, honeydew, nenas	1 potong/ 1 cawan	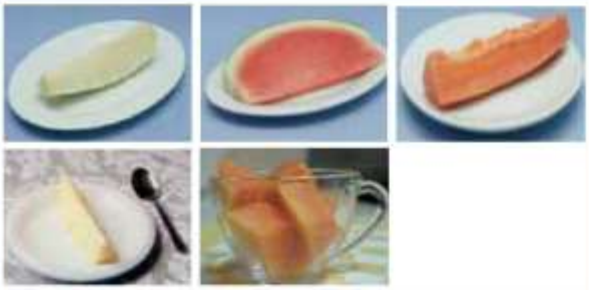
Rambutan, anggur, jambu air, duku, dokong	8 biji sederhana	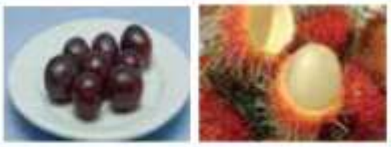
Pisang Emas	2 biji	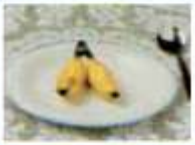
Buah nangka	3 ulas sederhana/ 4 ulas kecil	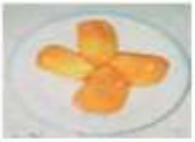
Durian	3 ulas	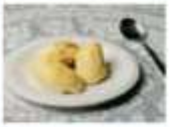

BUKU KOD I *CODE BOOK* 13

Kismis	1 sudu besar	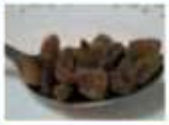
**B. SAYUR-SAYURAN**		
**SAYURAN HIJAU MENTAH/ ULAM-ULAMAN** Contoh: ulam-ulaman (daun ceylom, pegaga, raja, tomato, jering, jantung pisang, tomato, kacang botor)	= 1 cawan ulam-ulaman/ sayur mentah	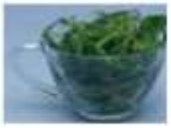
**SAYUR-SAYURAN LAIN, MASAK ATAU CINCANG** Contoh: bayam, kobis, brokoli, sawi	= ½ cawan sayur berdaun hijau dengan batang yang boleh dimakan seperti bayam, kangkung yang dimasak ATAU<br>= ½ cawan sayur buah yang dimasak (terung, lobak merah, labu dan tomato)<br>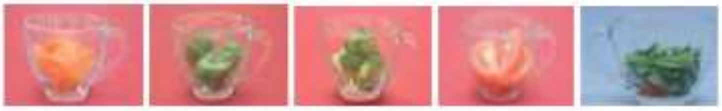<br>Atau 1 senduk<br>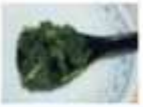<br>Atau 3 sudu makan<br>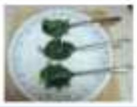	

Contoh Satu Gelas Air Kosong

Jenis	1 Gelas	Contoh
Gelas minuman	1gelas – 250ml	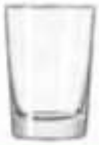
